# Incidence of Suspected Serious Adverse Drug Reactions in Corona Virus Disease-19 Patients Detected by a Pharmacovigilance Program by Laboratory Signals in a Tertiary Hospital in Spain: Cautionary Data

**DOI:** 10.3389/fphar.2020.602841

**Published:** 2020-12-03

**Authors:** Elena Ramírez, Mikel Urroz, Amelia Rodríguez, Miguel González-Muñoz, Alberto Martín-Vega, Yuri Villán, Enrique Seco, Jaime Monserrat, Jesús Frías, Antonio J. Carcas, Alberto M. Borobia

**Affiliations:** ^1^ Department of Clinical Pharmacology, La Paz University Hospital-IdiPAZ, School of Medicine, Autonomous University of Madrid, Madrid, Spain; ^2^ Department of Immunology, La Paz University Hospital-IdiPAZ, Madrid, Spain; ^3^ CSUR Coordination, La Paz University Hospital-IdiPAZ, Madrid, Spain; ^4^ Safety and Quality Unit, La Paz University Hospital-IdiPAZ, Madrid, Spain

**Keywords:** adverse drug reaction, corona virus disease-19 (COVID-19), serious adverse drug reaction, pharmacovigilance process, adverse (side) effects

## Abstract

**BACKGROUND:** From March to April 2020, Spain was the center of the SARS-CoV-2 pandemic, particularly Madrid with approximately 30% of the cases in Spain. The aim of this study is to report the suspected serious adverse drug reactions (SADRs) in COVID-19 patients vs. non-COVID-19 patients detected by the prospective pharmacovigilance program based on automatic laboratory signals (ALSs) in the hospital (PPLSH) during that period. We also compared the results with the suspected SADRs detected during the same period for 2019.

**METHODS:** All ALSs that reflected potential SADRs including neutropenia, pancytopenia, thrombocytopenia, anemia, eosinophilia, leukocytes in cerebrospinal fluid, hepatitis, pancreatitis, acute kidney injury, rhabdomyolysis, and hyponatremia were prospectively monitored in hospitalized patients during the study periods. We analyzed the incidence and the distribution of causative drugs for the COVID-19 patients.

**RESULTS:** The incidence rate of SADRs detected in the COVID-19 patients was 760.63 (95% CI 707.89–816.01) per 10,000 patients, 4.75-fold higher than the SADR rate for non-COVID-19 patients (160.15 per 10,000 patients, 95% CI 137.09–186.80), and 5.84-fold higher than the SADR rate detected for the same period in 2019 (130.19 per 10,000 patients, 95% CI 109.53–154.36). The most frequently related drugs were tocilizumab (59.84%), dexketoprofen (13.93%), azithromycin (8.43%), lopinavir-ritonavir (7.35%), dexamethasone (7.62%), and chloroquine/hydroxychloroquine (6.91%).

**CONCLUSIONS:** The incidence rate of SADRs detected by the PPSLH in patients with COVID-19 was 4.75-fold higher than that of the non-COVID-19 patients. Caution is recommended when using medications for COVID-19 patients, especially drugs that are hepatotoxic, myotoxic, and those that induce thromboembolic events.

## Introduction

From March to April 2020, Spain was the center of the severe acute respiratory syndrome coronavirus 2 (SARS-CoV-2) pandemic, (Center for Coordination of Health Alerts and Emergencies) with Madrid accounting for approximately 30% of the cases in Spain. (Current situation in the Community of Madrid) In response, the Spanish Ministry of Health published protocols for the care and management of COVID-19 patients. Azithromycin, chloroquine, hydroxychloroquine and lopinavir/ritonavir have been recommended for treatment during the infectious phase. Subsequently, anti-inflammatory drugs (such as corticosteroids and other compounds) were recommended for COVID-19 patients who progressed to the inflammatory phase of the disease. The drugs employed during that period were off-label or under development as potential treatment options for COVID-19.

In this situation, more attention should be paid to the safety of these drugs, whose toxicity profile is relatively well understood based on trials and the post-marketing experience in the indications for which they are approved. (Spanish Agency for Medicines and Health Products; Spanish Agency for Medicines and Health Products; Spanish Agency for Medicines and Health Products; Spanish Agency for Medicines and Health Products; Spanish Agency for Medicines and Health Products; Spanish Agency for Medicines and Health Products) Although the most frequent reactions to these drugs are usually mild, serious adverse effects have also been reported with their use. It is also unclear whether the use of these drugs by COVID-19 patients poses greater risks, because COVID-19 itself could be a predisposing factor to certain serious adverse drug reactions (SADRs).

In recent decades, large, computerized clinical databases linked to electronic medical records (EMRs) have helped implement prospective programs for detecting SADRs and aiding clinicians in reacting quickly and appropriately to these reactions. (Hannan, 1999) Since 2007, our hospital has employed a prospective pharmacovigilance program based on the systematic detection of predefined abnormal laboratory signals (ALSs) through our laboratory information system (Pharmacovigilance Program from Laboratory Signals in Hospital [PPLSH]) for the early detection of SADRs. The screening for specific anomalous laboratory data enables us to monitor a large number of patients with limited resources, thereby accessing high-quality information in a timely manner. (Ramirez et al., 2010) We conducted a thorough evaluation of ALSs during the current pandemic to help detect those events associated with the treatments and the disease and provide a basis for decision making in drug risk management during a possible second wave of the pandemic.

The aim of the study was to report the suspected SADRs in COVID-19 patients vs. non-COVID-19 patients detected by the PPLSH from March to April 2020. We also compared the results with the suspected SADRs detected during the same period for the previous year.

## Materials and Methods

### Setting

La Paz University Hospital in Madrid, Spain, is a tertiary-care teaching facility, where all admissions to wards are monitored by the PPLSH. The program was conducted according to the Spanish Personal Data Protection Law, (Organic law 3/2018, december 5, 2018, protection of personal data and guarantee of digital rights (BOE núm. 294, 119788-11985) and approval for publishing the program was obtained from the Institutional Review Board at La Paz University Hospital (protocol PI-3226). The technical document of the Spanish Ministry of Health for the clinical management of COVID-19 in emergencies and in hospital was adapted and implemented in the hospital. (Spanish Ministry of Health; World Health Organization).

### Information System and Coverage

A specific database application was developed within the integrated laboratory system (Labtrack Integrated Laboratory System), available in the hospital since 2003, which we employed to collect the predefined ALSs. We reviewed all ALSs retrieved systematically from these patients’ medical records.

At the time of the study, all of the patients’ medical information was collected in the hospital’s EMRs and included all laboratory data, imaging and other exploratory results, previous medical reports, medication prescription record, comments on the patients’ progression, and discharge summaries. Discharge summaries were coded according to International Statistical Classification of Diseases and Related Health Problems tenth revision (ICD-10).

Hospital laboratories that conduct blood tests for inpatients and emergency patients are certified and accredited under the appropriate International Standards Organization (ISO 9001:2000 and ISO 15189).

### Definition of Signals

Table 1 lists the criteria for selecting the drug-induced ALS.

**TABLE 1:** Definition of automatic laboratory signals used to detect serious adverse drug reactions.

Agranulocytosis
Neutrophils <0.5 × 10^3^/µl, hemoglobin ≥10 g/dl, platelets ≥ 100 × 10^3^/µl
Pancytopenia
White blood cells ≤ 3.5 × 10^3^/µl, hemoglobin ≤10 g/dl, platelets ≤ 50 × 10^3^/µl
Thrombocytopenia
Platelets <20 × 10^3^/µl, white blood cells >3.5 × 10^3^/µl, hemoglobin >10 g/dl
Anemia
Hemoglobin <6.5 g/dl
Eosinophilia
Eosinophils >0.8 × 10^3^/with organ involvement or systemic symptoms
Leukocytosis in the cerebrospinal fluid
Leukocytes ≥ 10 mm^3^
Liver injury
ALAT x 5 ULN IU/L
Pancreatitis
Amylase x 3 ULN IU/L or lipase x 3 ULN
Acute kidney injury
Creatinine x 3 ULN
Hyponatremia
Sodium ≤122 mmol/L
Rhabdomyolysis
Creatine kinase x 5 ULN IU/L

ALAT, alanine aminotransferase; ULN, upper limit of normal.

### Definitions of Adverse Drug Reaction

We employed the E2D definition of SADR of the International Council for Harmonisation of Technical Requirements for Pharmaceuticals for Human Use. (ICH Guideline on E2D Postapproval Safety Data Management: Definitions and Standards for Expedited Reporting) For the program’s purposes, we excluded ADRs caused by accidental or intentional overdose, as well as medical errors, which we considered to be any error in the written prescription, dispensation or administration. Errors in decision making (use in contraindicated clinical conditions or drug interactions) were considered SADRs and were therefore included. Adverse reactions caused by chemotherapy drugs were excluded from hematological ALSs, given that agranulocytosis, anemia, pancytopenia and thrombocytopenia are expected and explained by the pharmacodynamic properties of these drugs.

### Detecting and Evaluating Adverse Drug Reactions

**FIGURE 1:**
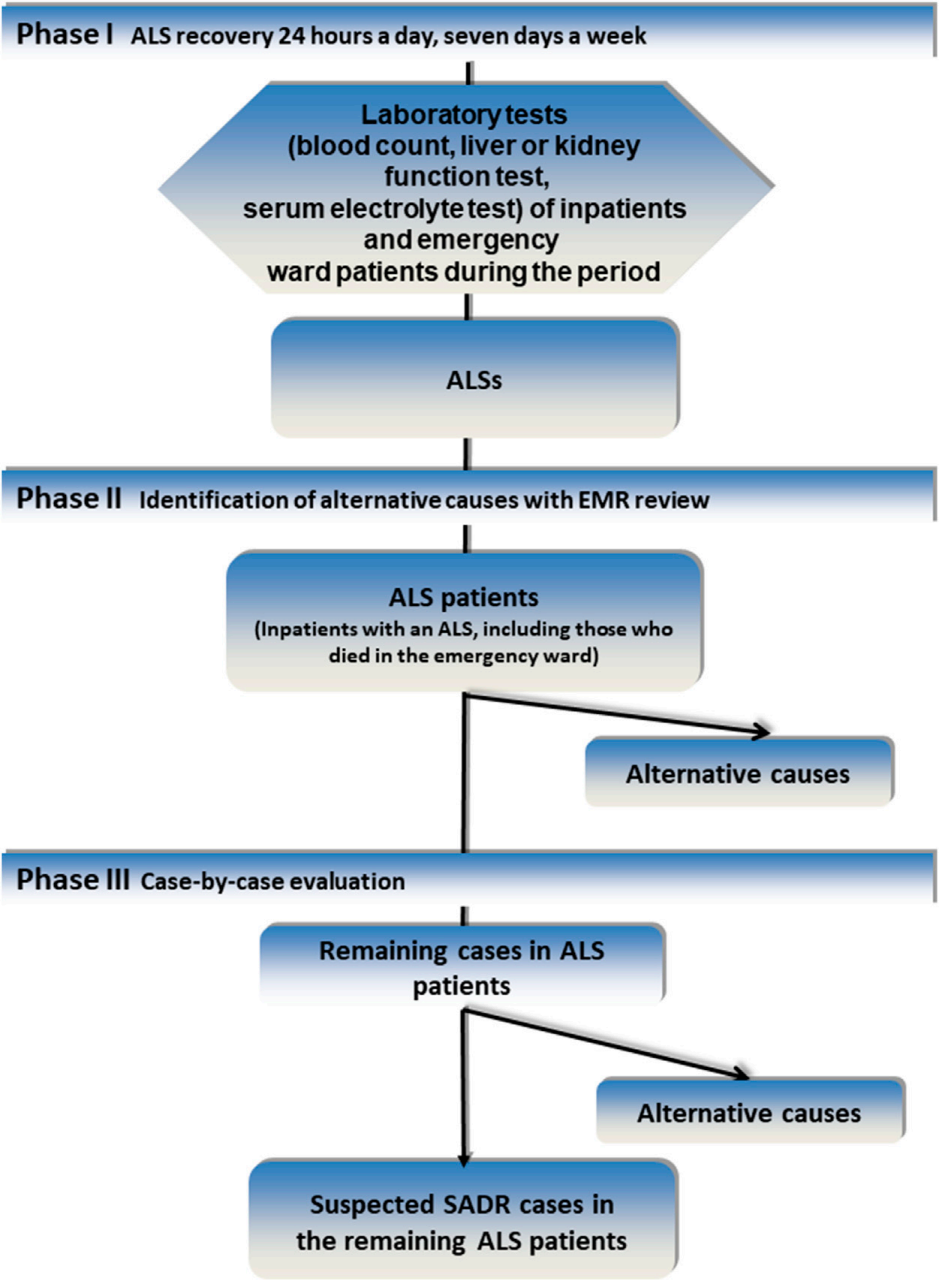
Methodology of the Pharmacovigilance Program From Laboratory Signals in Hospital. Abbreviations: ALS, automatic laboratory signal; EMR, electronic medical record; SADR, serious adverse reaction.

The procedure for detecting and evaluating ADRs has been described elsewhere. (Yelehe-Okouma et al., 2018) Briefly, in **phase I**, on-file laboratory data at admission or during hospitalization were screened 7 days a week, 24 h a day, for ALS from March to April 2019 and for the same period in 2020. In **phase II,** the patients were identified to avoid duplicates, and EMRs were reviewed. In **phase III**, a case-by-case evaluation was performed for the remaining cases (Figure 1). The causality assessment was performed using the algorithm of the Spanish Pharmacovigilance System. (Aguirre and García, 2016) We considered the categories of possible, probable or definite for drug-related reactions. Regarding the evaluation of the drug cause vs. the alternative cause (nondrug-induced), we only considered a drug cause when there was no alternative cause to explain the signal and, for the COVID-19 patients, when there was a dissociation between the clinical and lab parameters for improvement but a worsening of the ALS in the evaluation.

### Monitoring COVID-19 Patients

During hospitalization, patients with COVID-19 infection were monitored by their physicians to assess hepatic and renal function (alanine aminotransferase), aspartate aminotransferase, alkaline phosphatase, creatinine, gamma-glutamyl transferase, glomerular filtration rate, bilirubin, prothrombin activity and thromboembolic risk (hemoglobin, Chronic Kidney Disease Epidemiology Collaboration equation, D-dimer, fibrinogen, platelets). The lab controls varied depending on the patients’ clinical situation, usually daily during the first three weeks. We evaluated the laboratory test results for each drug for treating COVID-19 using the following structure: The baseline value was the value before drug administration; value 1 was the first value after administration; values 2–19 were the values on days 1–20 after the first dose administration; and values 20–39 were the results between days 21 and 60 after dose administration during the hospitalization.

### Collection of Patient Data and Reporting

For all patients initially categorized as having a suspected SADR, a complete report was submitted to the pharmacovigilance center in Madrid (https://www.notificaram.es).

### Data Analysis

The results are presented using central tendency measures (mean for quantitative variables and median for ordinal ones) and measures of dispersion (standard deviation and interquartile range, respectively) and percentages (95% confidence interval) for discrete variables. The in-hospital incidence rate for each SADR and other etiologies of ALS were calculated by dividing the number of cases of drug-induced reactions in hospitalized or deceased patients in the emergency department by the number of patients hospitalized during the selected months. We assessed the uncertainty of association by calculating the 95% two-sided Poisson confidence interval. The chi-squared test was performed to compare sex distribution and morbidity variables, and Student’s t-test or the Mann Whitney test, as appropriate, was used to compare the ages of patients in the SADR cohorts. We employed IBM SPSS Statistics for Windows, Version 20.0 (IBM Corporation, Armonk, NY, US) for the statistical analysis.

## Results

**FIGURE 2:**
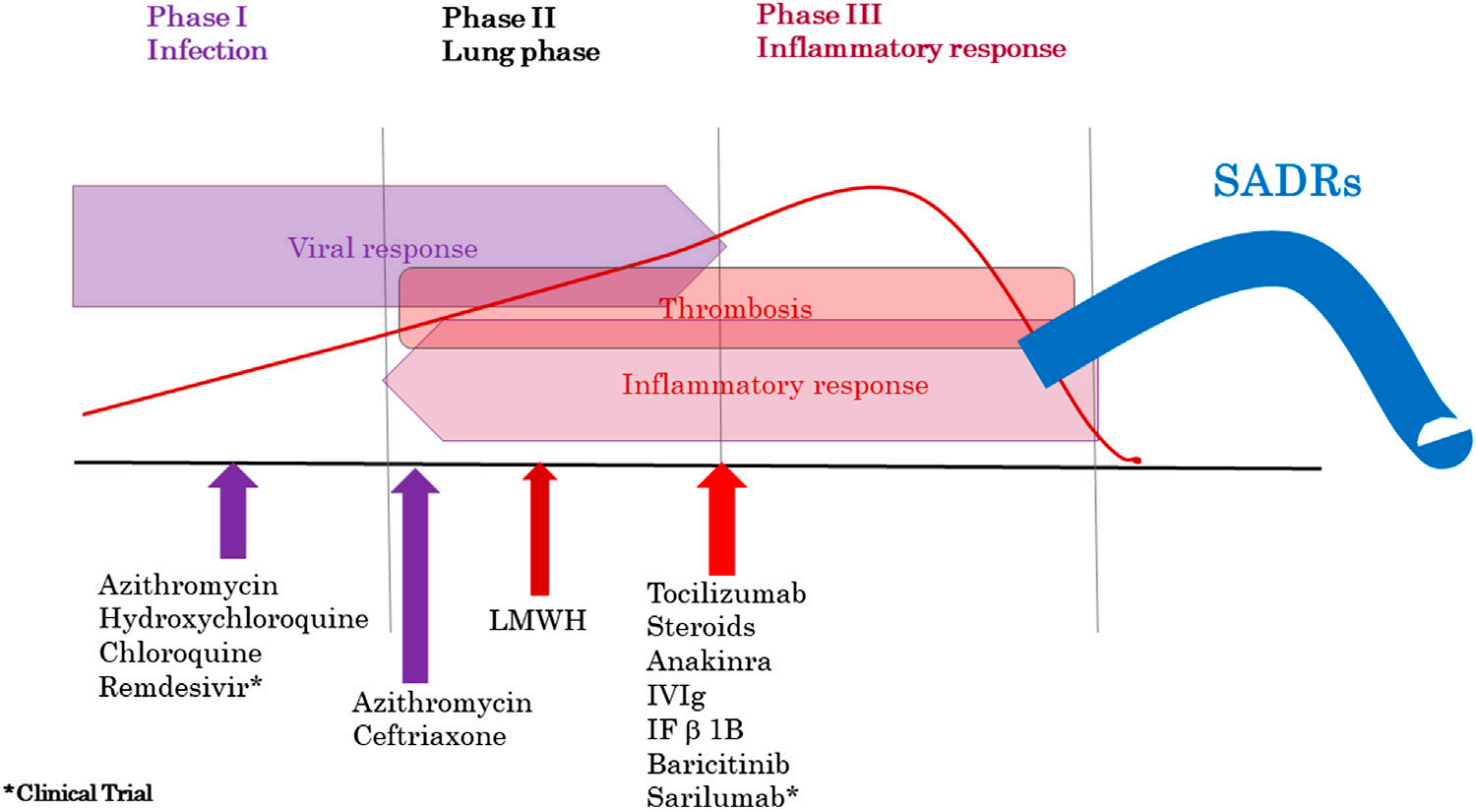
COVID-19 infection stages and treatments employed. Abbreviations: LMWH, low-molecular-weight heparin; Ig, immunoglobulin; IV Ig, intravenous immunoglobulin; IF *β*-1b, interferon beta-1b; SADRs, serious adverse drug reactions.

A total of 7,365 patients were hospitalized from March 1 to April 30, 2020, 2,682 (36.4%) of whom had COVID-19 infection. Figure 2 shows the sequence of drugs administered in the various phases of COVID-19 disease. The number of cases with ALS during the period was 1,341, with 575 COVID-19 patients and 766 non-COVID-19 patients. The COVID-19 patients had fewer hematological, pancreatitis and hyponatremia ALSs but more hepatitis, acute kidney injury, and rhabdomyolysis ALSs (Table 2). There were 1,153 cases with ALSs in the same period for 2019. The patients with COVID-19 in 2020 had an overall 3-fold higher rate of suspected SADRs than the non-COVID-19 patients in 2020 and 2019 (35.5% vs. 9.8% and 9.2%, respectively), with the following rates of SADRs: pancytopenia (57.1% vs. 1.4% and 4.5%), agranulocytosis (50% vs. 2.8% and 14.7%), thrombocytopenia (100% vs. 6.3% and 5.9%), anemia (43.8% vs. 3.2% and 27.6%), eosinophilia (14.1% vs. 5.7% and 4.1%), leukocytes in the cerebrospinal fluid (50% vs. 20.8% and 5.4%), hepatitis (45.1% vs. 23.7% and 12.4%), pancreatitis (58.3% vs. 15.6% and 7%), acute kidney injury (21.4% vs. 1.0% and 8.8%), rhabdomyolysis (15.3% vs. 4.4% and 9.5%) and hyponatremia (94.4% vs. 25.0% and 27.8%) (Table 2). The incidence rate of suspected SADRs detected by PPLSH in the COVID-19 patients was 760.63 (95% CI 707.89–816.01) per 10,000 patients, 4.75-fold higher than the SADR rate in the non-COVID-19 patients for the same period (160.15 per 10,000 patients, 95% CI 137.09–186.80) and 5.84-fold higher than the SADR rate for the same period in 2019 (130.19, 95% CI 109.53–154.36) (Table 3). The description of the demographic characteristics and morbidities of the patients who had SADRs during the study periods are shown in Table 4. Non-COVID-19 patients with SADRs were significantly older and there were significantly more females that COVID-19 patients, but the morbidities did not show significant differences between cohorts. Table 5 lists the drugs that most frequently produced SADRs in the COVID-19 patients, which includes: tocilizumab (59.84%), dexketoprofen (13.93%), azithromycin (8.43%), lopinavir-ritonavir (7.35%), dexamethasone (7.62%), and chloroquine/hydroxychloroquine (6.91%). The overall mortality rate for the COVID-19 vs. the non-COVID-19 patients (in 2020 and 2019) was 21.6% vs. 3.6% and 3.0%, respectively. The mortality rate for the COVID-19 patients with SADRs vs. the non-COVID-19 patients with SADRs (in 2020 and 2019) was 30.5% vs. 3.9% and 3.3%, respectively. Table 5 lists the mortality rate per drug for COVID-19 patients with SADRs.

**TABLE 2:** Breakdown by no SADR group vs. SADR group recorded from March to April 2020 in COVID-19 patients vs. non-COVID-19 patients.

Date	March to April 2020					
Cohort	COVID-19 patients					
Signal category	No SADR		SADR		Total Signals	
	*n*	%	*n*	%	*n*	**%**
Pancytopenia^a^	3	42.9	4	57.1	7	8.9
Agranulocytosis^a^	6	50.0	6	50.0	12	25.0
Thrombocytopenia^a^	0	0.0	2	100.0	2	11.1
Anemia^a^	9	56.3	7	43.8	16	20.5
Eosinophilia	67	85.9	11	14.1	78	30.8
Leukocytes CSF	1	50.0	1	50.0	2	7.7
Hepatitis	141	54.9	116	45.1	257	62.2
Pancreatitis	5	41.7	7	58.3	12	21.1
AKI III	88	78.6	24	21.4	112	52.1
Rhabdomyolysis	50	84.7	9	15.3	59	56.7
Hyponatremia	1	5.6	17	94.4	18	36.0
TOTAL	371	64.5	204	35.5	575	42.9

Date	March to April 2020					
Cohort	Non-COVID-19 patients					
Signal category	No SADR		SADR		Total Signals	
	*n*	%	*n*	%	*n*	**%**
Pancytopenia^a^	71	98.6	1	1.4	72	91.1
Agranulocytosis^a^	35	97.2	1	2.8	36	75.0
Thrombocytopenia^a^	15	93.8	1	6.3	16	88.9
Anemia^a^	60	96.8	2	3.2	62	79.5
Eosinophilia	165	94.3	10	5.7	175	69.2
Leukocytes CSF	19	79.2	5	20.8	24	92.3
Hepatitis	119	76.3	37	23.7	156	37.8
Pancreatitis	38	84.4	7	15.6	45	78.9
AKI III	102	99.0	1	1.0	103	47.9
Rhabdomyolysis	43	95.6	2	4.4	45	43.3
Hyponatremia	24	75.0	8	25.0	32	64.0
TOTAL	691	90.2	75	9.8	766	57.1

Date	March to April 2019					
Cohort	All ALS patients					
Signal category	No SADR		SADR		Total Signals	
	*n*	%	*n*	%	*n*	**%**
Pancytopenia^a^	84	95.5	4	4.5	88	7.6
Agranulocytosis^a^	29	85.3	5	14.7	34	2.9
Thrombocytopenia^a^	16	94.1	1	5.9	17	1.5
Anemia^a^	42	72.4	16	27.6	58	5.0
Eosinophilia	347	95.9	15	4.1	362	31.4
Leukocytes CSF	35	94.6	2	5.4	37	3.2
Hepatitis	184	87.6	26	12.4	210	18.2
Pancreatitis	53	93.0	4	7.0	57	4.9
AKI III	155	91.2	15	8.8	170	14.7
Rhabdomyolysis	76	90.5	8	9.5	84	7.3
Hyponatremia	26	72.2	10	27.8	36	3.1
TOTAL	1,047	90.8	106	9.2	1,153	100.0

Breakdown by no SADR group vs. SADR group recorded from March to April 2019. AKI, acute kidney injury; ALS, automatic laboratory signals; CSF, cerebrospinal fluid; IR, incidence rate per 10,000 patients; SADR, serious adverse drug reaction.

a SADRs by antineoplastic agents are in the no ADR group.

**TABLE 3:** Incidence rate (Poisson 95% CI) per 10,000 patients of no SADR vs. SADR recorded from March to April 2020 in COVID-19 patients vs. non-COVID-19 patients.

Date	March to April 2020					
COVID-19 cohort	COVID-19 patients					
Signal category	No SADR			SADR		
	IR	95% CI		IR	95% CI	
Pancytopenia^a^	11.19	6.20	19.68	14.91	8.40	23.50
Agranulocytosis^a^	22.37	14.58	33.31	22.37	14.58	33.31
Thrombocytopenia^a^	0.00	0.0	3.7	7.46	3.45	14.42
Anemia^a^	33.56	23.55	46.34	26.10	17.80	38.10
Eosinophilia	249.81	219.97	281.93	41.01	30.27	55.62
Leukocytes CSF	3.73	1.09	8.77	3.73	1.09	8.77
Hepatitis	525.73	482.00	571.89	432.51	393.17	474.72
Pancreatitis	18.64	11.44	28.45	26.10	17.79	38.10
AKI III	328.11	294.41	365.49	89.49	72.37	109.52
Rhabdomyolysis	186.43	161.16	214.74	33.56	23.55	46.34
Hyponatremia	3.73	1.09	8.77	63.39	49.29	80.60
TOTAL	1,383.30	1,312.04	1,457.86	760.63	707.89	816.01

Date	March to April 2020					
COVID-19 cohort	Non-COVID-19 patients					
Signal category	No SADR			SADR		
	IR	95% CI		IR	95% CI	
Pancytopenia^a^	151.61	128.80	177.10	2.14	0.62	7.22
Agranulocytosis^a^	74.74	58.99	92.90	2.14	0.62	7.22
Thrombocytopenia^a^	32.03	22.72	45.17	2.14	0.62	7.22
Anemia^a^	128.12	107.70	152.19	4.27	1.62	10.24
Eosinophilia	352.34	317.13	390.76	21.35	13.79	32.11
Leukocytes CSF	40.57	29.42	54.47	10.68	5.50	18.40
Hepatitis	254.11	224.66	287.23	79.01	62.44	98.46
Pancreatitis	81.14	65.22	100.68	14.95	8.40	23.49
AKI III	217.81	190.02	247.87	2.14	0.62	7.22
Rhabdomyolysis	91.82	74.17	111.73	4.27	1.63	10.24
Hyponatremia	51.25	38.83	67.06	17.08	10.67	27.22
TOTAL	1,475.55	1,401.65	1,552.24	160.15	137.09	186.80

Date	March to April 2019					
COVID-19 cohort	All ALS patients					
Signal category	No SADR			SADR		
	IR	95% CI		IR	95% CI	
Pancytopenia^a^	328.31	294.41	365.49	4.91	1.63	10.24
Agranulocytosis^a^	164.16	140.78	191.11	6.14	2.81	13.06
Thrombocytopenia^a^	72.96	57.22	90.67	1.23	0.24	5.57
Anemia^a^	282.71	251.0	316.91	19.65	12.22	29.67
Eosinophilia	797.98	743.59	854.31	18.42	11.44	28.45
Leukocytes CSF	109.44	90.41	131.49	2.46	0.62	7.22
Hepatitis	711.34	660.66	765.24	31.93	21.89	44.00
Pancreatitis	205.19	178.83	235.07	4.91	1.63	10.24
AKI III	469.67	428.46	513.43	18.42	11.44	28.45
Rhabdomyolysis	205.19	178.83	235.07	9.83	4.80	17.08
Hyponatremia	145.92	123.28	170.62	12.28	6.92	20.96
TOTAL	3,492.85	3,378.11	3,609.78	130.19	109.53	154.36

Incidence rate of no ADR vs. ADR recorded from March to April in 2019. AKI, acute kidney injury; ALS, automatic laboratory signals; CSF, cerebrospinal fluid; IR, incidence rate per 10,000 patients; SADR, serious adverse drug reaction.

a SADRs by antineoplastic agents are in the no ADR group.

**TABLE 4:** Characteristics of the patients who had SADRs during the study periods.

	SADRs in COVID-19 patients (*N* = 204)	SADRs in Non-COVID-19 patients in 2020 (*N* = 75)	*p* value^a^	SADRs in patients in 2019 (*N* = 106)	*p* value^b^
Number, median age, years (range)					
Adults^c^	201, 69.8 (21–96)	63, 74 (20–95)	<0.037	93, 75 (26–101)	<0.001
Children	3, 11 (9–14)	12, 7 (1–16)	—	13, 5.5 (0.1–16)	—
Sex, *n* (%, 95% CI)					
Male	151 (74, 66.4–81.6)	30 (40, 30.9–49.8)	<0.001	46 (43.4, 33.7–52.8)	<0.001
Female	53 (26, 18.4–35.4)	45 (60, 50.2–69.1)	—	60 (56.6, 46.2–65.3)	—
Comorbidities, *n* (%, 95% CI)					
Arterial hypertension	83 (40.7, 30.9–49.8)	30 (40.0, 30.9–49.8)	1.000	42 (39.6, 30–48.8)	0.880
Chronic heart disease	15 (7.5, 3-4–13.7)	11 (14.3, 8.5–22.1)	0.110	16 (15.1, 9.3–23.3)	0.071
Diabetes mellitus	36 (17.6, 10-9–25.5)	16 (21.3, 14.2–30)	0.470	23 (21.7, 14.2–30)	0.470
Rheumatological disease	3 (1.5, 0.2–5.4)	2 (2.0, 0.6–7)	0.560	2 (1.5, 0.2–5.4)	1.000
Solid malignant disease	11 (5.4, 2.2–11.2)	5 (6.1, 2.8–12.5)	0.760	7 (6.6, 2.8–12.5)	0.760
Obesity	13 (6.3, 2.8–12.5)	6 (8.2, 4.1–15)	0.580	8 (7.5, 3.4–13.7)	0.770
Chronic kidney disease	14 (6.9, 2.8–12.5)	9 (12.0, 7–19.8)	0.140	13 (12.1, 7–19.8)	0.140
Chronic obstructive pulmonary disease	22 (10.8, 5.5–17.4)	12 (16.0, 10.1–24.4)	0.210	17 (16.0, 10.1–24.4)	0.210
Other chronic lung disease	13 (6.3, 2.8–12.5)	5 (6.1, 2.8–12.5)	1.000	8 (7.5, 3.4–13.7)	0.770
Hematological malignant disease	4 (2.0, 0.6–7)	3 (4.1, 1.6–9.8)	0.410	5 (4.7, 1.6–9.8)	0.410
Asthma	9 (4.4, 1.6–9.8)	3 (4.0, 1.6–9.8)	1.000	5 (4.5, 1.6–9.8)	1.000
Liver disease	12 (5.9, 2.2–11.2)	8 (9.3, 4.8–16.2)	0.270	10 (8.5, 4.1–15)	0.390
HIV Infection	1 (0.6, −0 to 3.7)	1.3 (2.0, 0.6–7)	0.160	2 (1.5, 0.6–5.4)	0.320

CI, confidence level.

a COVID-19 patients vs. Non-COVID-19 patients 2020.

b COVID-19 patients vs. Patients 2019.

c ≥18 years old.

**TABLE 5:** Medications, prescriptions, comorbidities, outcome, serious adverse drug reactions.

Medication	Patients	Age, years	Sex (Male)	Liver disease	Chronic kidney disease	Heart disease	ICU	Outcome	Duration, days	Dosage, mg/day	SADR
	*n*	Mean (SD)	%	%	%	%	%	1. Home, %2. deceased, %3. Transferred, %4. Voluntary discharge, %	Median (IQ)	Median (IQ)	Signal (*n*)
Azithromycin	1,008	66.8 (15.8)	57.6	4.3	6.5	1.8	6.4	1. 70.0	3 (1–12)	500 (400–600)	Total (85), AA (1) E (2) H (74) PC (1) NA (2) Tp (5)
								2. 22.7			
								3. 7.0			
								4. 0.2			
Chloroquine - hydroxychloroquine	1924	67.3 (17.7)	56.8	3.9	7.2	6.0	5.4	1. 68.7	4.37 (1–36)	151 (100–800)	Total (133), AA (3) A (3) T (1) E (7) MEN (1) H (107) PC (2) R (1) NA (6) Tp (2)
								2. 24.0			
								3. 7.1			
								4. 0.2			
Ceftriaxone	1,118	69.9 (17.5)	58.9	4.5	8.9	7.7	6.4	1. 63.5	4 (1–18)	2,000 (800–4,000)	Total (48), AA (1), A (1) E (2), H (43) NA (1)
								2. 29.3			
								3. 7.0			
								4. 0.2			
Levofloxacin	345	68.7 (16.9)	58.4	4.0	8.8	7.5	5.4	1. 63.8	4 (1–17)	500 (400–800)	Total (6), A (1) AN (1) H (2) PC (1) NA (1)
								2. 29.0			
								3. 7.0			
								4. 0.2			
Lopinavir/ritonavir	245	65.9 (15.8)	58.8	4.9	4.5	6.1	9.8	1. 62.9	4 (1–14)	800/200 (400/100–1,600/400)	Total (18), MEN (1) H (13) PC (3) AKI (1)
								2. 31.0			
								3. 5.7			
								4. 0.4			
Dexketoprofen	122	62.2 (16.9)	54.3	3.6	16	10.2	7.0	1. 78.9	5 (1–36)	50 (25–75)	Total (17), T (1) AN (1) H (3) AKI (12)
								2. 14.0			
								3. 7.0			
								4. 0.0			
Metamizole	1,548	66.3 (17.7)	55.2	4.3	7.4	6.8	8.9	1. 68.0	8 (1–23)	3,000 (1,000–6,000)	Total (9), AN (3) E (1) H (4) PC (1) AKI (1)
								2. 25.6			
								3. 6.1			
								4. 0.3			
Paracetamol (acetaminophen)	2,357	67.7 (18.1)	55.5	4.3	8.4	7.8	6.6	1. 66.6	8 (1–44)	3,000 (500–3,000)	Total (18), AN (1) H (17)
								2. 26.7			
								3. 6.6			
								4. 0.2			
LMWH	2,206	68.5 (16.2)	55.6	4.0	8.0	7.4	6.3	1. 68.0	9 (1–51)	9 (1–51)	Total (29), AN (13) E (7) MEN (1) H (8)
								2. 25.1			
								3. 6.7			
								4. 0.2			
Dexamethasone	210	62.9 (20.5)	44.3	6.6	7.4	6.6	20.5	1. 46.7	7 (1–22)	11 (4–40)	Total (16), AN (5) H (2) PC (5) R (3) NA (1)
								2. 48.4			
								3. 4.9			
								4. 0.0			
Tocilizumab	127	61.1 (11.02)	68.5	3.9	1.6	1.6	22.8	1. 68.5	1 (1–5)	440 (0–800)	Total (76), A (2) AN (2) E (7) H (54) PC (2) R (1) Fib (8)
								2. 26.8			
								3. 3.1			
								4. 1.6			

AA, aplastic anemia; A, agranulocytosis; AKI, acute kidney injury; AN, anemia; E, eosinophilia with organ involvement or systemic symptoms; Fib, hypofibrinogenemia; H, hepatitis; IQ, interquartile; LMWH, low molecular weight heparin; MEN, meningitis; NA, hyponatremia; PC, pancreatitis; R, rhabdomyolysis; SADRs, serious adverse drug reactions; T, thrombocytopenia; Tp, troponin I.

**FIGURE 3:**
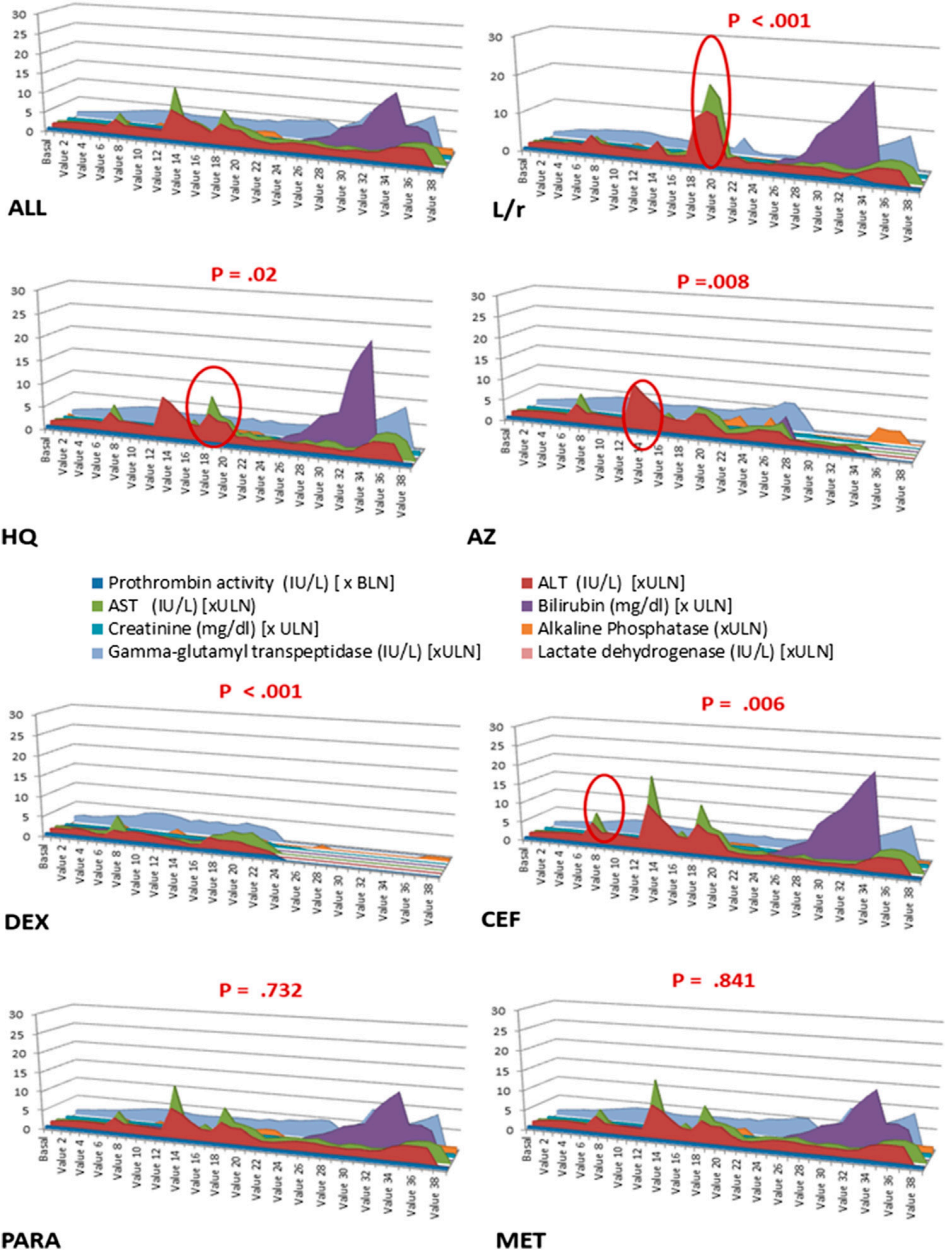
Sequence chart: Progression of parameters related to liver function in COVID-19 patients during hospitalization according to the treatment undergone. The *X*-axis indicates the value number (baseline, the last value before drug administration; value 1, first value after administration; values 2–19, values on days 1–20 after the first dose administration; values 20–39, results between days 21 and 60 of hospitalization). The *Y*-axis indicates the number of times above or below the limit of normality, as appropriate. The red circle indicates the points with statistical significance. ALL, all patients; AZ, azithromycin; BLN, below limit of normal; CEF, ceftriaxone; HQ, hydroxychloroquine; DEX, dexamethasone, L/r, lopinavir/ritonavir; MET, metamizole; PARA, paracetamol; ULN, upper limit of normality.

**FIGURE 4:**
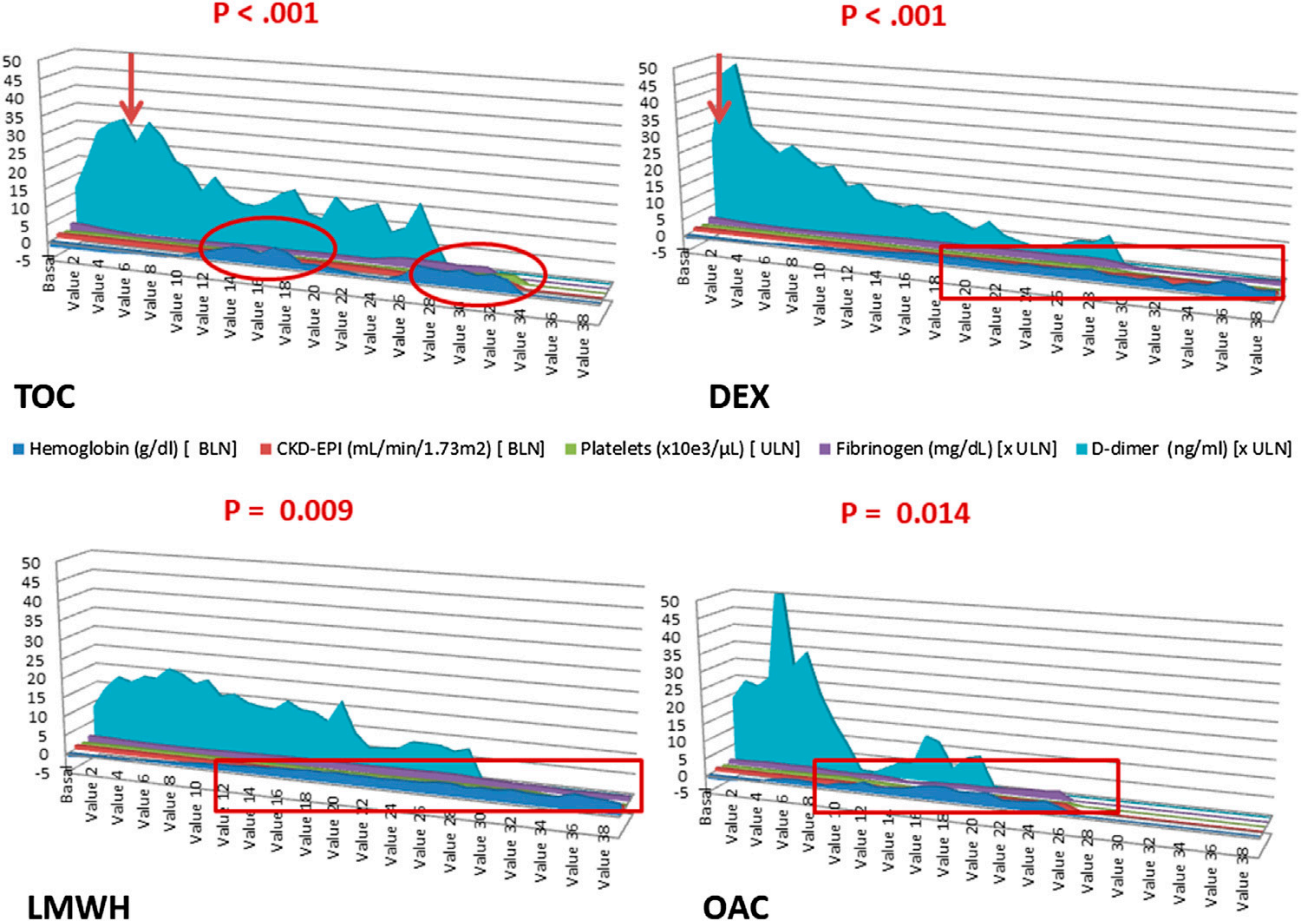
Sequence chart: Progression of parameters related to coagulation status in COVID-19 patients during hospitalization according to the treatment undergone. The *X*-axis indicates the value number (baseline, the last value before drug administration; value 1, first value after administration; values 2–19, values on days 1–20 after the first dose administration; values 20–39, results between days 21–60 of hospitalization). The *Y*-axis indicates the number of times above or below the limit of normality, as appropriate. The arrows indicate the change in the trend of normality. The red circles indicate the points with statistical significance. BLN, below limit of normal; DEX, dexamethasone; LMWH, low-molecular-weight heparins; OAC, oral anticoagulants; TOC, tocilizumab; ULN, upper limit of normal.

At the time of hospitalization, the COVID-19 patients had slightly abnormal liver function, based on alanine aminotransferase and gamma-glutamyl transferase levels that were slightly above the upper limit of normality (ULN), which were normal in most patients. Figure 3 shows the statistically significant worsening of liver function in the COVID-19 patients associated with the drugs used in phase I of the infection. Liver function parameters in the patients who took ceftriaxone typically increased by the end of the first week, in patients who took azithromycin increased in the second week, and in the patients who took lopinavir-ritonavir or hydroxychloroquine increased in the third week. However, liver function trended towards normalization during hospitalization for the patients who took dexamethasone. Metamizole and paracetamol showed no statistically significant effect. At the time of hospitalization, the COVID-19 patients had an increased thromboembolic risk measured by the number of fold increases in D-dimer levels above the ULN. Figure 4 shows the worsening of D-dimer, fibrinogen, and hemoglobin levels in the patients treated with the various drugs during phase II of the infection. Tocilizumab and dexamethasone produced a statistically significant reduction in fibrinogen and hemoglobin levels, along with a significant increase in D-dimer levels. Oral anticoagulants, dexamethasone and low-molecular-weight heparins were associated with a decrease in hemoglobin levels after the start of the drug in this hospitalization phase.

## Discussion

Since 2007, physicians of the Clinical Pharmacology Department of our hospital maintain uninterruptedly a pharmacovigilance program based on laboratory signals using its available information systems. ALSs were chosen on the basis that they were detectable in the routine tests of almost all laboratories and were therefore easily detectable in inpatients and because the ALSs could warn of relevant SADRs with significant impact on patient health and wellbeing. Agranulocytosis, aplastic anemia, eosinophilia, liver injury, and rhabdomyolysis are frequently evaluated ALSs in the literature. (Risks of agranulocytosis and aplastic anemia. A first report of their relation to drug use with special reference to analgesics. The International Agranulocytosis and Aplastic Anemia Study, 1986; Ramírez et al., 2017; Andrade et al., 2005; Wen et al., 2019) Thrombocytopenia, anemia, leukocytes in cerebrospinal fluid, pancreatitis, acute kidney injury, and hyponatremia are increasingly frequent drug-induced reactions. (Aster and Bougie, 2007; Carnovale et al., 2015; Yelehe-Okouma et al., 2018; Wu and Huang, 2018; Ramírez et al., 2019; Zheng et al., 2019) This study enabled us to detect a relevant number of ALSs that are potentially related to SADRs and to determine their in-hospital incidence. During March-April 2020, there was a significant 72% reduction in the number of spontaneous SADRs (not from the PPLSH) compared with the same period in 2019, although 4 drug reactions with eosinophilia and systemic symptoms, 2 cases of Stevens Johnson syndrome, and 1 case of acute generalized exanthematous pustulosis were included. In addition, we considered a spontaneous SADR as a sentinel event that motivated a root cause analysis from the hospital, which included, as an improvement action, a drug safety note for the Spanish Agency for Medicine and Health Products.

Overall, we detected a 5.8-fold higher rate of SADRs in the COVID-19 patients than during the same period of the previous year. The use of off-label medicines has been associated with more ADRs than the use of labeled medicines. (Bellis et al., 2014; Viola et al., 2016; Auffret et al., 2017)^,^ This off-label use would be acceptable if the evidence of potential benefits outweighs the ADR risk. More than 150 clinical trials are currently underway to study drugs that prevent or treat COVID-19 infection, some of which have shown no benefit, such as lopinavir-ritonavir, azithromycin, chloroquine and hydroxychloroquine. (Cao et al., 2020; Rosenberg et al., 2020; Tang et al., 2020a) During the COVID-19 pandemic period, we detected more SADRs in the non-COVID-19 patients than during the same period in 2019, an effect that can be explained by the overloading of the health system, which resulted in a patient safety problem: changes in prescription due to supply problems, insufficient deprescribing, more empiric treatments, and drug interactions in non-COVID-19 patients.

The PPLSH detected that 35.5% of the ALSs in the COVID-19 patients were SADRs, which agrees with the results from the China Hospital Pharmacovigilance System that detected ADRs in 37.8% of COVID-19 patients, which were predominately drug-induced gastrointestinal disorders and liver disorders (23.0% vs. 13.8% respectively). (Sun et al., 2020) Drug-induced liver injury was the most frequent SADR detected (116/204, 56.86%) in the study. Liver damage in mild cases of COVID-19 is often transient, and liver function can return to normal without special treatment. (Alqahtani and Schattenberg, 2020) Moderate and severe liver damage could be drug-induced, which might explain the large variation in liver impairment observed across the various cohorts. (Aggarwal et al., 2020; Borobia et al., 2020; Chen et al., 2020; Guan et al., 2020; Zhou et al., 2020) Immune-mediated inflammation, such as cytokine storms and pneumonia-associated hypoxia, might also have contributed to liver injury and even to the development of liver failure in critically ill patients with COVID-19. Figure 2 shows the increase in bilirubin and transaminase levels in the patients hospitalized for more than 4 weeks, corresponding to the critically ill patients.

Increased D-dimer levels have been reported as one of the most common laboratory findings in COVID-19 patients requiring hospitalization. (Tang et al., 2020b) D-dimer levels on admission 4-fold higher than the ULN have been associated with in-hospital mortality for patients with COVID-19. (Zhang et al., 2020) Despite the difficulties in standardizing D-dimer levels, test kit manufacturers, normal values and units, D-dimer is a marker of thromboembolic disease and disseminated intravascular coagulation. (Favaloro and Thachil, 2020) Drugs for treating phase II of infection-associated hyperinflammatory syndrome that can cause life-threatening acute respiratory distress syndrome in patients with COVID-19 pneumonia can also cause thromboembolic disease. These drugs include dexamethasone, baricitinib, sarilumab, interferon beta 1B, and intravenous immunoglobulins. (Spanish Agency for Medicines and Health Products; Spanish Agency for Medicines and Health Products; Spanish Agency for Medicines and Health Products; Spanish Agency for Medicines and Health Products) Thrombocytopenia has been associated with anakinra, and hypofibrinogenemia has been related to tocilizumab. (Spanish Agency for Medicines and Health Products; Spanish Agency for Medicines and Health Products) Our study therefore reports a statistically significant effect of hypofibrinogenemia with an increase in D-dimer levels associated with tocilizumab and to a lesser extent with dexamethasone. Anemization was also observed in the patients treated with low-molecular-weight heparins or oral anticoagulants during this phase of COVID-19.

This study’s main limitation is that the evaluation of causality of a possible SADR does not completely exclude the influence of COVID-19. Considering the current evidence, some overlap with COVID-19 cannot be ruled out. However, a drug-related cause was only considered when there was a dissociation between clinical improvement and worsening of the ALS. Longer follow-up periods are needed to assess the recovery or sequelae of these SADRs and to study the immunological and pharmacogenetic mechanisms and the re-exposure effects of these SADRs.

In conclusion, PPLSH has been useful in detecting and evaluating specific SADRs during the avalanche of hospitalizations of patients with COVID-19. The incidence rate of SADRs detected by PPSLH in the patients with COVID-19 was 4.75-fold higher than that of the non-COVID-19 patients. Caution is recommended in using drugs to treat patients with COVID-19, because the drugs cause additional damage, especially those that are hepatotoxic, myotoxic, or induce thromboembolic events.

## Data Availability Statement

The raw data supporting the conclusions of this article will be made available by the authors, without undue reservation.

## Ethics Statement

The studies involving human participants were reviewed and approved by Institutional Review Board at La Paz University Hospital (protocol PI-3226). Written informed consent for participation was not provided by the participants’ legal guardians/next of kin because: The requirement for informed consent was waived because the data collection was retrospective. All suspected ADRs were notified to the Spanish Pharmacovigilance System.

## Author Contributions

Participated in research design: ER and AMB. Data curation: MU, AR, YV, and ES. Participated in data analysis: JM, AM-V, and MG-M. Participated in the writing of the paper: ER, MU, and AMB. Writing – review and editing: MG-M, JF, and AC.

## Conflict of Interest

The authors declare that the research was conducted in the absence of any commercial or financial relationships that could be construed as a potential conflict of interest.
